# Assessing patient satisfaction among COVID-19 survivors in Northeast India: a cross-sectional study

**DOI:** 10.3389/fmed.2024.1308288

**Published:** 2024-05-30

**Authors:** Shazina Saeed, Farah Niazi, Karuna Nidhi Kaur, Shweta Rana, Manmohan Singhal, Harpreet Singh

**Affiliations:** ^1^Amity Institute of Public Health and Hospital Administration, Amity University, Noida, India; ^2^Faculty of Pharmacy, School of Pharmaceutical and Population Health Informatics, DIT University, Dehradun, India; ^3^Laboratory of Disease Dynamics & Molecular Epidemiology, Amity Institute of Public Health and Hospital Administration, Amity University, Noida, India; ^4^Division of Biomedical Informatics (BMI), Indian Council of Medical Research, New Delhi, India

**Keywords:** patient satisfaction, COVID-19 survivors, healthcare services, Northeast India, healthcare delivery

## Abstract

**Introduction:**

This study investigates patient satisfaction among COVID-19 survivors in Northeast India, motivated by the unique long-term healthcare needs of survivors and the critical role of patient satisfaction in assessing and enhancing healthcare quality. By focusing on this underexplored region, the research aims to uncover insights that can guide improvements in patient-centered care and healthcare service delivery in similar contexts.

**Materials and methods:**

The study was conducted using a Simple Random Sampling technique. Data were collected through telephone interviews using a semi-structured questionnaire, including the Patient Satisfaction Questionnaire-18 (PSQ-18) for analysis. The PSQ-18 yielded seven subscale scores representing different dimensions of patient satisfaction. Statistical analysis using SPSS software was conducted to summarize socio-demographic characteristics, medical history, and patient satisfaction levels, employing both descriptive and inferential statistics.

**Result:**

The results indicated a high acceptance of COVID-19 vaccination, with the majority of participants having received both doses. Patient satisfaction with healthcare services is generally positive, particularly in aspects related to doctor-patient communication and medical care quality. However, there are notable concerns regarding the affordability and timeliness of care. Regional variations across states, as well as factors like education and income, significantly influence patient satisfaction levels.

**Conclusion:**

The study revealed generally good patient satisfaction levels in Northeast India. However, challenges in healthcare affordability and timeliness persist, influenced by regional disparities and socio-economic factors. Targeted interventions are needed to improve healthcare in the region.

## 1 Introduction

The COVID-19 pandemic has not only reshaped healthcare delivery and patient experiences globally but has also brought to the forefront the critical importance of patient satisfaction as a cornerstone of healthcare quality (1, 2). This is particularly relevant in regions like Northeast India, where the healthcare landscape was already fraught with challenges even before the onslaught of the pandemic. The area has long grappled with issues such as inadequate healthcare infrastructure, limited medical facilities, and a scarcity of healthcare professionals, which have compounded the difficulties faced by patients in accessing quality care (3).

These pre-existing challenges in Northeast India’s healthcare system underscore the need for a nuanced understanding of patient satisfaction, especially in the wake of COVID-19. The pandemic added layers of complexity to an already strained system, making the assessment of patient satisfaction among COVID-19 survivors not just a measure of healthcare quality but a critical indicator of the system’s capacity to withstand and adapt to unprecedented crises. In this context, patient satisfaction serves as a lens through which the resilience, adaptability, and responsiveness of healthcare services to the heightened demands of a global health emergency can be evaluated (4).

The scarcity of studies specifically focusing on the experiences of COVID-19 survivors in India’s intricate socio-cultural and healthcare setting highlights a significant gap in the literature. This gap is more pronounced in the context of Northeast India, where the unique confluence of socio-economic, geographical, and cultural dynamics necessitates a tailored approach to healthcare satisfaction research. Such an approach is vital not only for enhancing the understanding of healthcare quality in the region but also for informing policy-making that prioritizes patient-centric care in the aftermath of the pandemic (5).

The distinct challenges and experiences faced by COVID-19 survivors in Northeast India, ranging from the struggle to access overstretched healthcare services to navigating the socio-cultural nuances of illness and recovery in a region with diverse ethnicities and traditions, demand an in-depth exploration. This exploration is crucial for unveiling the layers of patient satisfaction and the multifaceted interactions between patients and the healthcare system during and after the pandemic (6).

This study, by focusing on the comprehensive assessment of patient satisfaction among COVID-19 survivors in Northeast India, aims to address these critical issues. It intends to employ a cross-sectional research design and use validated assessment tools to examine various dimensions of patient satisfaction, including but not limited to, overall satisfaction with care, the technical quality of healthcare services, interpersonal interactions with healthcare providers, and accessibility to medical facilities. The outcomes of this study are anticipated to contribute significantly to the existing body of knowledge, providing evidence-based insights that can guide the enhancement of healthcare delivery in the region.

Furthermore, by spotlighting patient satisfaction among COVID-19 survivors in Northeast India—a region already burdened with healthcare challenges prior to the pandemic—this research endeavors to fill a notable void in existing studies. It aims to offer a detailed examination of patient experiences and satisfaction levels, thereby facilitating the development of healthcare practices and policies that are not only effective but also culturally and socially attuned to the unique needs and challenges of the region in the post-pandemic era.

## 2 Materials and methods

The primary objective of this cross-sectional study is to evaluate patient satisfaction among COVID-19 survivors in the Northeast State of India. In the context outlined in the introductory section, our study focused on the Northeast region of India, a region where healthcare infrastructure is notably under-researched. This discrepancy has led to uneven and often unsatisfactory improvements in healthcare infrastructure. The COVID-19 pandemic further underscored these challenges, underscoring the importance of assessing patient satisfaction, levels related to the healthcare delivery in these states. The study was conducted from February and August, 2023. During this period, the pandemic’s dynamics, including the emergence of new variants, changes in transmission rates, and the impact of vaccination campaigns have evolved significantly. This allows for a comprehensive understanding of patient satisfaction across different epidemiological situations and thus providing insights into how these changes influence patient satisfaction.

The study population consisted of individuals aged 18 and above who were hospitalized for COVID-19 treatment in the Northeast State of India and were subsequently discharged. Participants were included if they provided informed consent, were residents of the Northeast State, and were above 18 years of age at the time of their hospital admission. Exclusion criteria included those who did not provide informed consent, were under 18 years of age at the time of admission, were deceased, or were residents of other states in India.

The study received ethical approval from the University of Amity Institutional Review Board (IRB No. AUUP/IEC/MAY/2023/4).

### 2.1 Study population and sampling technique

In this study, among the 4,500 participants approached, a total of 2,000 respondents completed the questionnaire, resulting in a response rate of 44.4%. We aimed for a 95% confidence level with a 0.5 standard deviation and a 5% margin of error, leading to an initial sample size of 385 per state, across 7 states, totaling 2,695 respondents. However, due to higher-than-expected non-response rates, we adjusted our target and concluded the study with 2,000 respondents, ensuring the study’s integrity and reliability despite the challenges.

The selection process involved a carefully compiled list of discharged COVID-19 patients, ensuring each individual had an equal chance of being selected, in line with simple random sampling principles. Participants were recruited through a telephonic survey, where they were thoroughly briefed on the study’s objectives and ethical considerations, including the significance of their participation. To maintain a uniform and accessible informed consent process through the telephonic survey, verbal consent was obtained from all participants. This approach was aimed to accommodate all participants, ensuring clear understanding and voluntary participation.

### 2.2 Data collection tool

A semi-structured questionnaire was prepared and divided into five segments: socio-demographic profile, medical history, vaccination status, details of hospitalization, and the Patient Satisfaction Questionnaire-18 (PSQ-18). The socio-demographic profile included details such as age, gender, household income, etc., whereas the section medical history included questions addressing pre-existing co-morbidity disorders and family medical history. Vaccination status included the details as to if the respondents were vaccinated and reasons for non-vaccination. Details of hospitalization included questions about the type of hospital and the duration of the hospital stay. The PSQ-18 is a validated instrument developed by Grant N. Marshall and Ron D. Hays in 1994, and it employs a 5-point Likert scale ranging from “Strongly Agree” to “Strongly Disagree.” This tool is globally recognized for its efficacy in measuring patient satisfaction, allowing for the results to be compared and accepted by the international research community (7). The PSQ-18 section of the questionnaire consisted of 18 items related to general satisfaction, technical quality, interpersonal manner, communication, financial aspects, time spent with doctor, accessibility, and convenience.

To enhance accessibility, the questionnaire was available in English and Hindi, respecting regional linguistic preferences. Primarily conducted in English, provisions were made for Hindi-speaking respondents to ensure inclusivity. The questionnaire’s validity was ensured through a rigorous translation and back-translation process by independent experts.

We have defined key variables to comprehensively assess patient satisfaction among COVID-19 survivors in Northeast India. Patient Satisfaction, our primary variable, is gauged through individuals’ perceived quality of care and services received during their hospitalization for COVID-19. This includes evaluating technical quality, interpersonal manner, communication, accessibility, and convenience, as measured by the Patient Satisfaction Questionnaire-18 (PSQ-18). The Healthcare Delivery Context variable captures the conditions and infrastructure surrounding healthcare service provision, such as hospital type, infrastructure quality, and healthcare provider availability. Patient Demographics are considered to include age, gender, household income, and other socio-demographic factors that could influence satisfaction levels. Furthermore, Vaccination Status is scrutinized to understand if pre-hospitalization COVID-19 vaccination, including the number of doses and vaccine type, impacts patient satisfaction. Medical History is also a critical variable, encompassing pre-existing comorbidities and health conditions that might influence a patient’s experience and satisfaction during hospitalization. These variables are framed within the Patient Satisfaction Model, which theorizes that satisfaction is influenced by the quality of healthcare delivery, patient-provider interactions, and the healthcare environment, with our study extending this model to incorporate the unique challenges posed by the COVID-19 pandemic in the context of Northeast India.

The inclusion of these particular variables—Patient Satisfaction, Healthcare Delivery Context, Patient Demographics, Vaccination Status, and Medical History—is crucial for providing a holistic understanding of the factors that influence patient satisfaction. These variables were carefully chosen to capture a comprehensive view of the patient experience, recognizing that satisfaction is not only a reflection of the immediate healthcare services received but also influenced by broader contextual factors such as demographic characteristics, the patient’s health background, and their vaccination status. This multi-dimensional approach is essential for identifying actionable insights that can improve healthcare delivery and patient experiences, especially in the challenging context of a global pandemic.

For assessing the questionnaire’s internal consistency, a Cronbach’s alpha coefficient of 0.96 was computed, indicating a high level of reliability.

Data was collected via telephone interviews, where the questionnaire was administered to the participants. All data will be anonymized to maintain confidentiality and will be stored securely.

### 2.3 Patient satisfaction outcomes/scoring

In the analysis of the Patient Satisfaction Questionnaire-18 (PSQ-18), the instrument was scored to yield seven distinct subscale scores, each representing a different dimension of patient satisfaction. These dimensions include General Satisfaction, Technical Quality, Interpersonal Manner, Communication, Financial Aspects, Time Spent with Doctor, and Accessibility and Convenience. It is noteworthy that the PSQ-18 contains items phrased both positively and negatively to capture varying levels of satisfaction or dissatisfaction. Regardless of the phrasing, all items were scored in a manner that a higher score consistently indicated greater satisfaction with medical care. Following the item-level scoring, the responses within each of the seven subscales were averaged to generate the respective subscale scores. In essence, each subscale score represents the mean score of all answered items within that specific dimension. This scoring methodology represent the average for all items in the scale that were answered.

In our study, we have identified patient satisfaction as the primary outcome of interest. The constructs of General Satisfaction, Technical Quality, Interpersonal Manner, Communication, Financial Aspects, Time Spent with Doctor, and Accessibility and Convenience were defined as exposure variables within the framework of this study. Additionally, demographic and socio-economic covariates, including age, gender, and socioeconomic status, were analyzed to ascertain their potential influence on the variability observed in patient satisfaction metrics.

### 2.4 Data analysis

Statistical analyses were conducted on the 2,000 collected data using SPSS software. Initial steps involved descriptive statistics to outline the basic features of the participants’ socio-demographic information, medical history, and their satisfaction with healthcare services. Further, inferential statistics were employed to discern patterns and significant connections in the data. Chi-square test was performed to assess the influence of various factors on patient satisfaction.

## 3 Results

The demographic analysis of our participant pool highlights significant diversity and varying characteristics. Predominantly, the age distribution skewed toward younger adults, with significant representation in the 18–25 and 26–33 age brackets. Gender distribution was notably skewed, with a higher proportion of male participants. A considerably high number of the respondents were married, and the predominant religion among participants was Hinduism, followed by other religions including Islam, Sikhism, Christianity, and Buddhism in smaller proportions.

Educational status among participants varied widely, yet a significant portion held graduate degrees. The occupational background was diverse, with a notable percentage of participants working in the healthcare sector. Most of the participants were employed in the private sector, reflecting a range of employment sectors within the study population. Household income levels were diverse, indicating a broad socio-economic representation among the participants (Table 1).

**Table 1:** Socio-demographic characteristics of the study participants.

	Variables	No. of participants	Percentage (%)
Age	<18 years	4	0.2
	18–25	290	14.5
	26–33	567	28.4
	34–40	401	20.1
	41–47	279	14
	48–55	201	10.1
	56–63	140	7
	>63 years	118	5.9
Gender	Female	481	24.1
	Male	1,517	75.9
	Others	2	0.1
Marital status	Married	1,532	76.6
	Single	454	22.7
	Divorced/Widowed	14	0.7
Religion	Hindu	1,752	87.6
	Muslim	146	7.3
	Sikhs	22	1.1
	Christian	60	3
	Buddhism	6	0.3
	Others	14	0.7
Education	Illiterate/uneducated	106	5.3
	Secondary	190	9.5
	Higher secondary	300	15
	Graduate	1,019	50.9
	Post-graduate and above	385	19.3
Occupation	Unemployed	108	5.4
	Student	106	5.3
	Security guards	2	0.1
	Police	14	0.7
	Healthcare workers	311	15.6
	Others	1,459	73
Employment sector	Government	532	26.6
	Private	840	42
	Self-employed	402	20.1
Household income (INR)	Less than 50,000	112	5.6
	50,000–1.5 lac	432	21.6
	1.6 lac-2.5 lac	719	35.9
	2.6 lac and above	737	36.9

**Figure 1:**
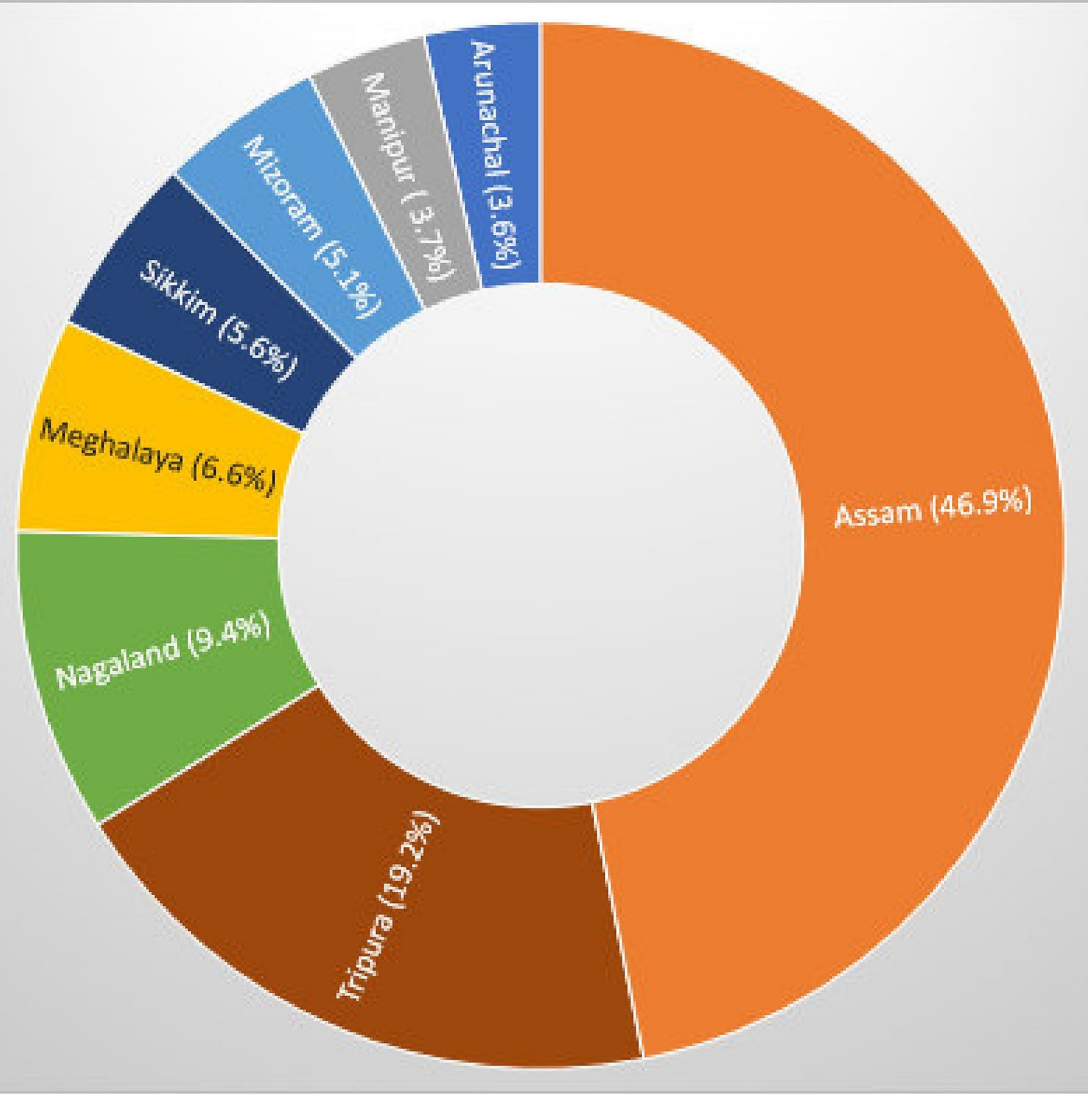
State-wise distribution of study participants.

Geographical distribution of the study participants showed a notable variance across regions, with Assam leading in participant representation, followed by a significant number from Tripura. Contributions from smaller states such as Manipur, Arunachal, and Sikkim were comparatively minimal (Figure 1).

Table 2 presents an overview of health-related behaviors and conditions among participants. It highlights the prevalence of alcohol and tobacco use, with most of those indicating no history of use in both categories. Additionally, the table details the presence of pre-existing co-morbidities and family medical history of diseases, showing a larger proportion of participants without such conditions or histories.

**Table 2:** Habits, pre-existing co-morbidities and family history of the study participants.

Variables			No. of participants	Percentage (%)
Habits	Use of alcohol	Current user	255	12.8
		Past user	68	3.4
		Never used	1,677	83.9
	Use of tobacco	Current user	220	11
		Past user	44	2.2
		Never used	1,736	86.8
Pre-existing co-morbidities		Yes	341	17.1
		No	1,659	83
Family medical history		Yes	323	16.1
		No	1,677	83.9

**Figure 2:**
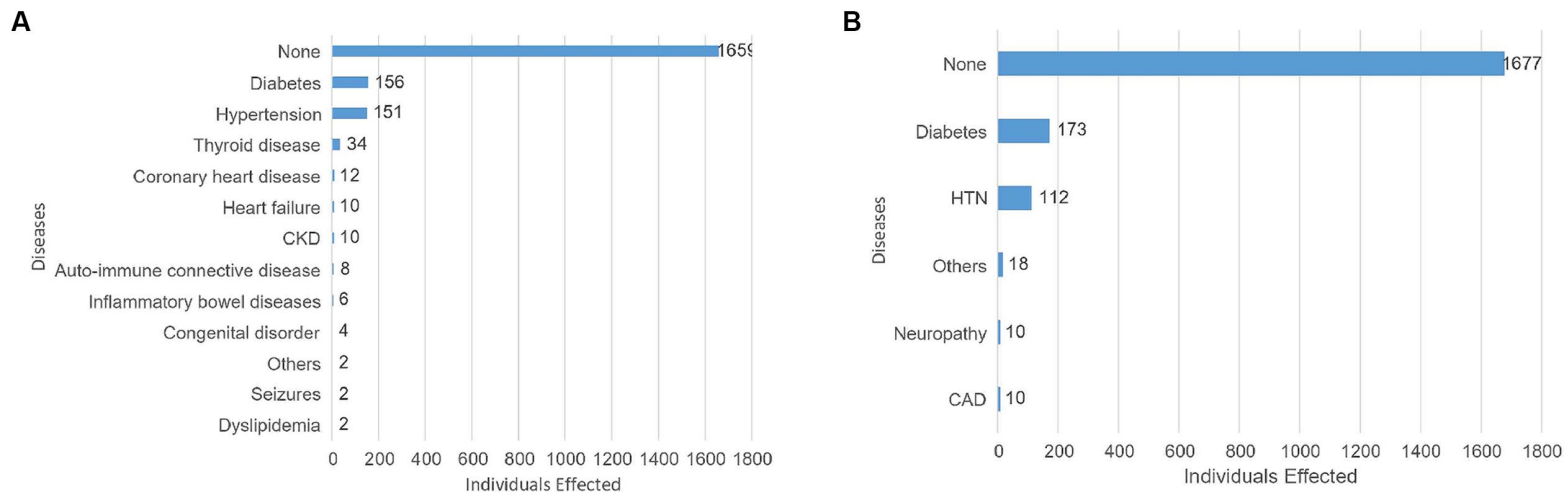
Pre-existing co-morbidities among study participants **(A)** and family medical history of study participants **(B)**.

The most common co-morbidities among the study participants were Diabetes and Hypertension. However, the vast number of participants, reported having no pre-existing conditions at all. Diabetes was also the most commonly reported family medical history condition, followed by Hypertension (HTN). However, a overwhelming number indicated that they have had no family history of medical conditions (Figure 2).

**Figure 3:**
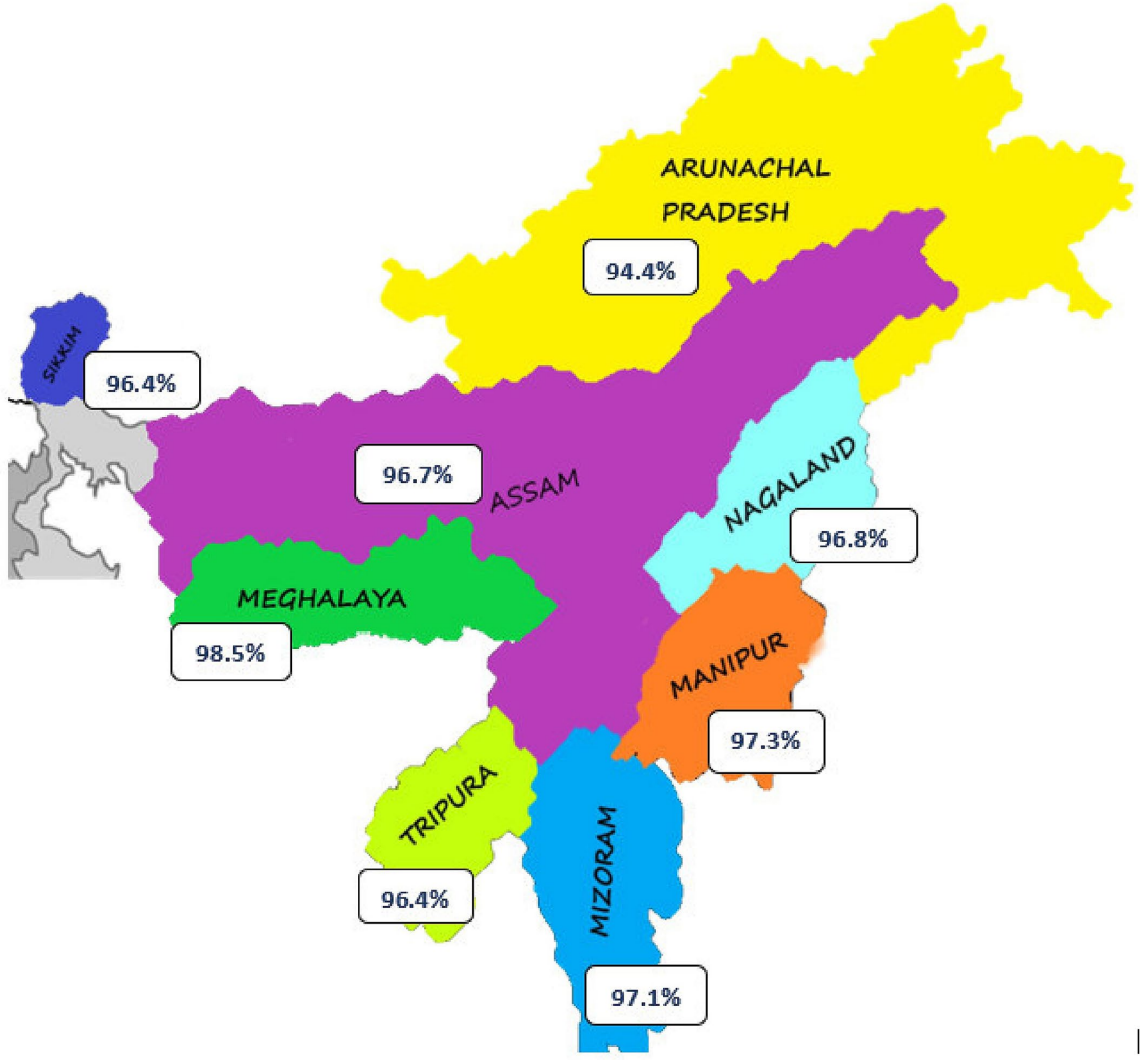
COVID-19 vaccination status across 8 north-eastern states as per the study findings.

The study revealed valuable insights about the COVID-19 vaccination status and related factors among the study’s participants. Notably, a significant proportion of participants had received their first dose of the COVID-19 vaccine. Additionally, a substantial portion had also completed their second dose. For the minority who had not been vaccinated, reasons ranged from a perceived lack of necessity to vaccine unavailability and medical contraindications. Geographic analysis revealed variations in vaccination rates across different states, with Arunachal Pradesh, Meghalaya, and Mizoram showing higher rates compared to Assam and Tripura (Table 3 and Figure 3).

**Table 3:** Vaccination status and hospitalization details of the study participants.

Variables		No. of participants	Percentage (%)
Received COVID-19 vaccine (Dose 1)	Yes	1,934	96.7
	No	66	3.3
Received COVID-19 vaccine (Dose 2)	Yes	1,892	94.6
	No	108	5.4
If no, reason for not receiving the vaccine	Didn’t feel the need to get vaccinated	56	28
	Fear of side effects	4	0.2
	Unavailability of vaccine	24	1.2
	Medical conditions	20	1
	Not aware about the vaccine	4	0.2
Type of hospital	Government	1,304	65.2
	Private	640	32
	Semi-government	56	2.8
Duration of stay in the hospital	<1 week	715	35.8
	1 week	522	26.1
	2 weeks	400	20
	3 weeks	293	14.7
	>3 weeks	70	3.5
Post COVID-19 complications	Acute cardiac injury	22	1.1
	Acute respiratory failure	26	1.3
	Anxiety, depression	72	3.6
	Asthma, breathlessness, chest pain	74	3.7
	Chronic fatigue, excessive weakness, excessive weight loss	74	3.7
	GI problems	18	0.9
	Pneumonia	40	2
	Loss of smell	28	1.4
	None	1,646	82.3

The analysis of healthcare settings indicated that a large number of participants were treated in government hospitals, followed by private and semi-government facilities. The duration of hospital stays also showed a range, with many participants having shorter stays of less than 1 week, and a decreasing proportion staying for longer periods, up to more than 3 weeks.

The study also provides information on post-COVID-19 complications. While a significant number of the study participants reported no complications following recovery, a subset experienced various issues, including acute cardiac injury, acute respiratory failure, mental health challenges such as anxiety and depression, respiratory symptoms like asthma and chest pain, chronic fatigue, gastrointestinal problems, pneumonia, and loss of smell (Table 3).

**Figure 4:**
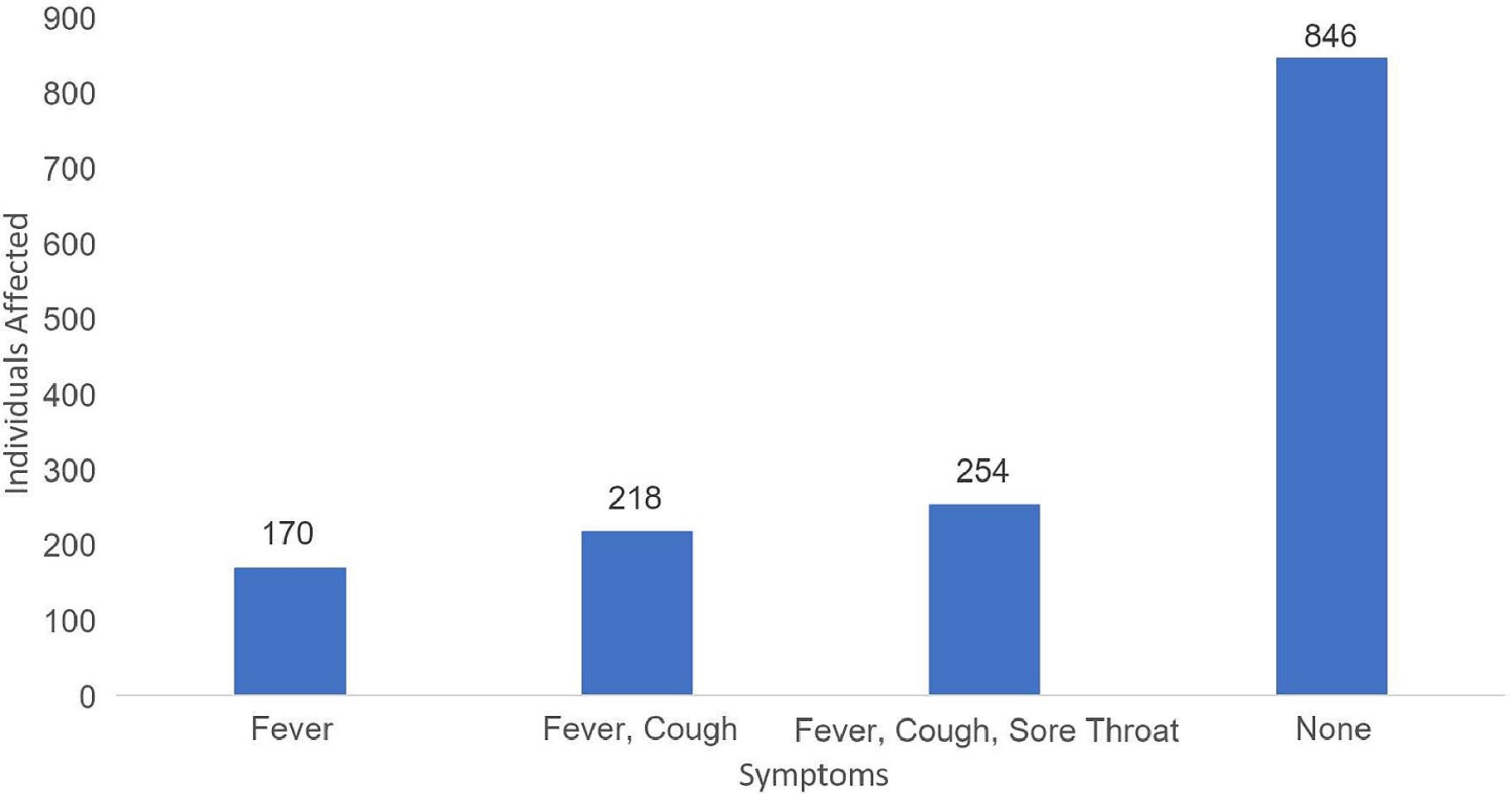
Major clinical symptoms reported in the study participants.

The most frequently reported clinical symptom combination was “Cough, Sore throat, Fever.” This was followed by “Cough, Fever” and isolated “Fever.” Interestingly, a significant portion of the study population reported experiencing no symptoms (Figure 4).

**Figure 5:**
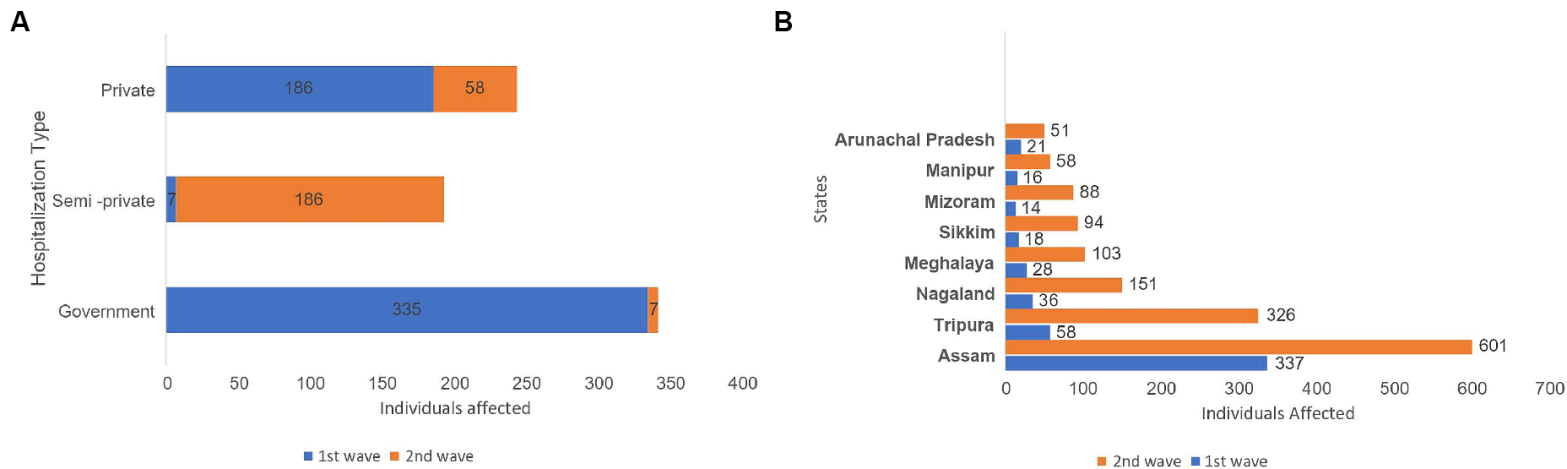
Graphical representation of the difference in the incidence of hospitalized COVID-19 patients between the first and second waves **(A)**, and patient distribution across the 8 northeastern states **(B)** analyzed using a Chi-square test for statistical significance.

The study analyzed the relationship between how patients are distributed across the eight northeastern states and the different types of hospitalizations, and the results were found to be statistically significant (Figure 5).

Based on the responses to the Short-Form Patient Satisfaction Questionnaire (PSQ-18), most patients had a positive perception of their medical care. They felt doctors explained medical tests well, their doctor’s office was well-equipped, and the care they received was almost perfect. However, financial concerns and long wait times for emergency treatment were areas of concern. Additionally, some patients felt doctors were impersonal or ignored their concerns, indicating room for improvement in doctor-patient interactions (Table 4).

**Table 4:** Short-form Patient Satisfaction Questionnaire (PSQ-18).

Questions (Item numbers)	Strongly agree	Agree	Uncertain	Disagree	Strongly disagree	Original response value	Scored value
1. Doctors are good about explaining the reason for medical tests.	261 (13.1%)	**1,382 (69.1%)**	40 (2.0%)	202 (10.1%)	115 (5.8%)	2	4
2. I think my doctor’s office has everything needed to provide complete medical care.	272 (13.6%)	**1,385 (69.3%)**	44 (2.2%)	200 (10.0%)	99 (5.0%)	2	4
3. The medical care I have been receiving is just about perfect.	274 (13.7%)	**1,349 (67.5%)**	46 (2.3%)	225 (11.3%)	106 (5.3%)	2	4
4. Sometimes doctors make me wonder if their diagnosis is correct.	96 (4.8%)	246 (12.3%)	126 (6.3%)	**1,464 (73.2%)**	68 (3.4%)	4	4
5. I feel confident that I can get the medical care I need without being set back financially.	268 (13.4%)	**1,394 (69.7%)**	50 (2.5%)	202 (10.1%)	86 (4.3%)	2	4
6. When I go for medical care, they are careful to check everything when treating and examining me.	304 (15.2%)	**1,317 (65.9%)**	48 (2.4%)	229 (11.5%)	102 (5.1%)	2	4
7. I have to pay for more of my medical care than I can afford.	64 (3.2%)	183 (9.2%)	84 (4.2%)	**1,589 (79.5%)**	80 (4.0%)	4	4
8. I have easy access to the medical specialists I need.	284 (14.2%)	**1,333 (66.6%)**	54 (2.7%)	233 (11.7%)	96 (4.8%)	2	4
9. Where I get medical care, people have to wait too long for emergency treatment.	90 (4.5%)	279 (14.0%)	120 (6.0%)	**1,445 (72.3%)**	66 (3.3%)	4	4
10. Doctors act too businesslike and impersonal toward me.	106 (5.3%)	182 (9.1%)	74 (3.7%)	**1,544 (77.2%)**	94 (4.7%)	4	4
11. My doctors treat me in a very friendly and courteous manner.	226 (11.3%)	**1,379 (69.0%)**	90 (4.5%)	197 (9.9%)	108 (5.4%)	2	4
12. Those who provide my medical care sometimes hurry too much when they treat me.	104 (5.2%)	255 (12.8%)	74 (3.7%)	**1,491 (74.6%)**	76 (3.8%)	4	4
13. Doctors sometimes ignore what I tell them.	112 (5.6%)	215 (10.8%)	82 (4.1%)	**1,499 (75.0%)**	92 (4.6%)	4	4
14. I have some doubts about the ability of the doctors who treat me.	74 (3.7%)	158 (7.9%)	60 (3.0%)	**1,600 (80.0%)**	108 (5.4%)	4	4
15. Doctors usually spend plenty of time with me.	228 (11.4%)	**1,133 (56.7%)**	240 (12.0%)	270 (13.5%)	129 (6.5%)	2	4
16. I find it hard to get an appointment for medical care right away.	105 (5.3%)	234 (11.7%)	30 (1.5%)	**1,559 (78.0%)**	72 (3.6%)	4	4
17. I am dissatisfied with some things about the medical care I receive.	179 (9.0%)	182 (9.1%)	18 (0.9%)	**1,380 (69.0%)**	241 (12.0%)	4	4
18. I am able to get medical care whenever I need it.	268 (13.4%)	**1,363 (68.2%)**	38 (1.9%)	245 (12.3%)	86 (4.3%)	2	4
**Total score (Out of 90)**							**72**

Bold values are indicates all the statistically significant *p* values.

Table 5 demonstrates the PSQ-18 Sub-Scale Scores that provide a concise overview of patient satisfaction in various healthcare aspects. Patients reported an average score of 4 for General Satisfaction, Technical Quality, Interpersonal Manner, Communication, Financial Aspects, Time Spent with Doctor, and Accessibility and Convenience. This suggests an overall positive perception of healthcare experiences across these dimensions.

**Table 5:** PSQ-18 sub-scale scores.

Scale	Questions (Item numbers)	Average score
General satisfaction	3, 17	4
Technical quality	2, 4, 6, 14	4
Interpersonal manner	10, 11	4
Communication	1, 13	4
Financial aspects	5, 7	4
Time spent with doctor	12, 15	4
Accessibility and convenience	8, 9, 16, 18	4

**Figure 6:**
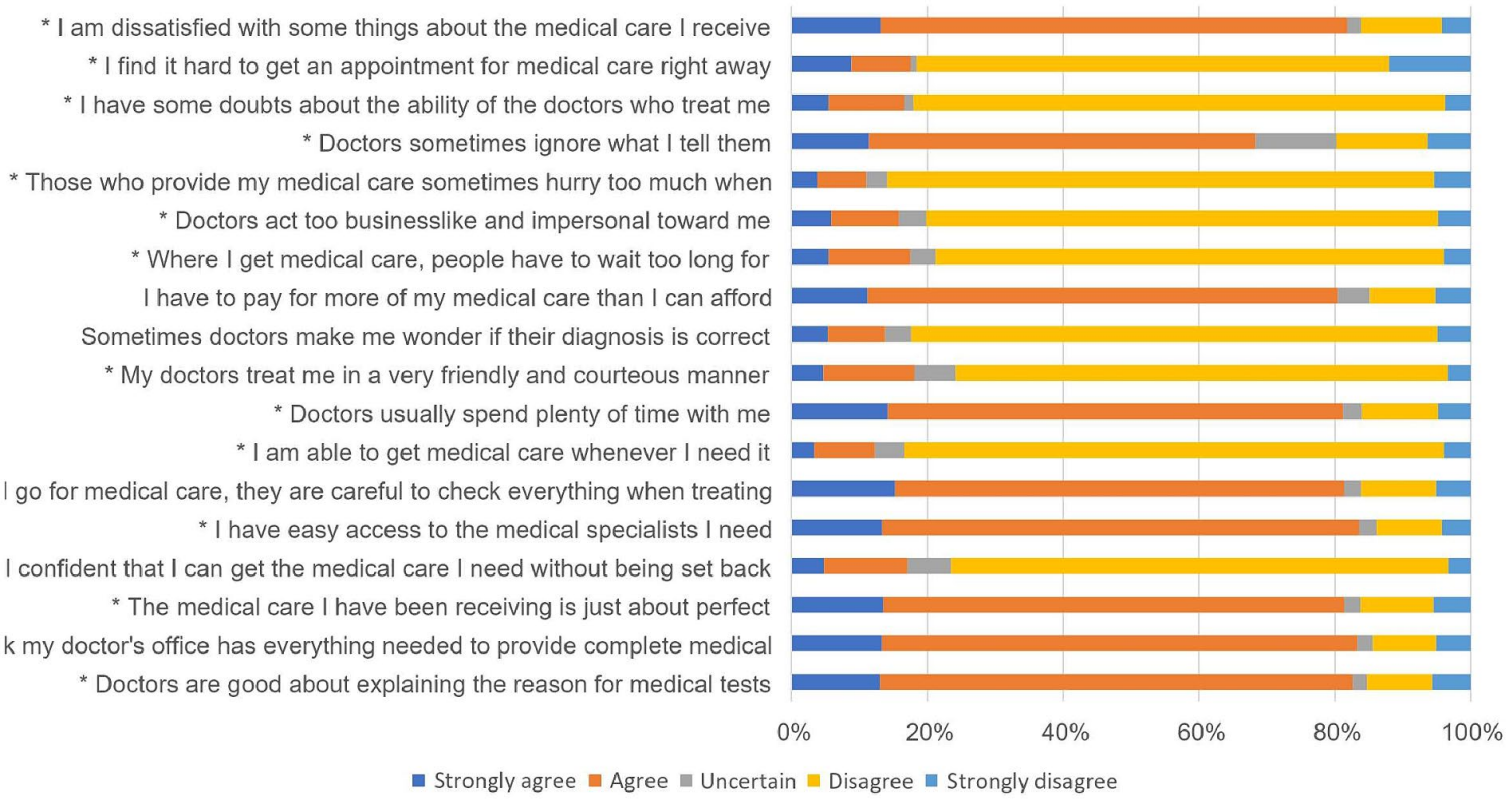
Bi-variate relationship between receiving of COVID-19 vaccine and patient satisfaction (Participants received the vaccine) (* = significant *p*-value).

Figure 6 elucidates the relationship between COVID-19 vaccination and patient satisfaction across multiple healthcare dimensions. High levels of satisfaction were observed in domains such as doctor-patient communication and medical care quality, with over 80% of participants agreeing or strongly agreeing. However, significant dissatisfaction was noted in areas like affordability and timeliness of care, where over 75% disagree or strongly disagree. Most observations were statistically significant with *p*-values below 0.05.

The study also examined the relationship between participants’ education status, household income, and states of residence in relation to patient satisfaction with various aspects of medical care using a chi-square test. The analysis showed significant variations across education, income, and states (Table 6). Upon analyzing the association between hospital type, patient stay duration, and patient satisfaction, findings consistently indicated high patient satisfaction across hospital types and stay durations, with almost all *p*-values showing strong statistical significance (Table 7).

**Table 6:** Bivariate relationships: education, household income, state of residence, and their impact on patient satisfaction.*

Questions (Item numbers) (see Appendix)		Education N (%)					Household income (Rs.) N (%)				States N (%)							
		Uneducated	Secondary	Higher secondary	Graduate	PG												
							<50,000	50,001–1.5 lac	1.6–2.5 lac	>2.6 lac	AP	As	Mn	Mg	Mz	Ng	Sk	Tr
Q1.	SA	10 (0.5)	22 (1.1)	40 (2.0)	151 (7.6)	38 (1.9)	4 (0.2)	30 (1.5)	156 (7.8)	71 (3.6)	2 (0.1)	129 (6.5)	2 (0.1)	20 (1.0)	2 (0.1)	24 (1.2)	10 (0.5)	72 (3.6)
	A	68 (3.4)	136 (6.8)	210 (10.5)	676 (33.8)	292 (14.6)	98 (4.9)	304 (15.2)	429 (21.5)	551 (27.6)	57 (2.9)	643 (32.2)	55 (2.8)	87 (4.4)	86 (4.3)	127 (6.4)	87 (4.4)	240 (12.0)
	U	0 (0.0)	6 (0.3)	4 (0.2)	22 (1.1)	8 (0.4)	2 (0.1)	14 (0.7)	12 (0.6)	12 (0.6)	0 (0.0)	2 (1.0)	1 (0.1)	5 (0.3)	1 (0.1)	3 (0.2)	0 (0.0)	10 (0.5)
	Di	26 (1.3)	18 (0.9)	34 (1.7)	102 (5.1)	22 (1.1)	8 (0.4)	68 (3.4)	82 (4.1)	44 (2.2)	8 (0.4)	93 (4.7)	10 (0.5)	10 (0.5)	12 (0.6)	25 (1.3)	10 (0.5)	34 (1.7)
	SD	2 (0.1)	8 (0.4)	12 (0.6)	68 (3.4)	25 (1.3)	0 (0.0)	16 (0.8)	40 (2.0)	59 (2.9)	5 (0.3)	53 (2.7)	6 (0.3)	9 (0.50)	1 (0.1)	8 (0.4)	5 (0.3)	28 (1.4)
	*p*-value	**<0.001**					**<0.001**				**<0.001**							
Q2.	SA	12 (0.6)	28 (1.4)	34 (1.7)	162 (8.1)	36 (1.8)	2 (0.1%)	30 (1.5%)	158 (7.9%)	82 (4.1)	14 (0.7)	129 (6.5)	7 (0.4)	14 (0.7)	3 (0.2)	22 (1.1)	12 (0.6)	71 (3.6)
	A	72 (3.6)	130 (6.5)	228 (11.4)	667 (33.4)	288 (14.4)	98 (4.9)	308 (15.4)	427 (21.3)	552 (27.6)	45 (2.3)	650 (32.5)	51 (2.6)	100 (5.0)	84 (4.2)	123 (6.2)	85 (4.3)	247 (12.4)
	U	2 (0.1)	6 (0.3)	8 (0.4)	26 (1.3)	4 (0.2)	2 (0.1)	10 (0.5)	16 (0.8)	16 (0.8)	0 (0.0)	22 (1.1)	0 (0.0)	2 (0.1)	3 (0.2)	6 (0.3)	3 (0.2)	8 (0.4)
	D	18 (0.9)	22 (1.1)	22 (1.1)	108 (5.4)	30 (1.5)	8 (0.4)	72 (3.6)	80 (4.0)	40 (2.0)	8 (0.4)	92 (4.6)	10 (0.5)	9 (0.5)	11 (0.5)	27 (1.4)	8 (0.4)	35 (1.8)
	SD	2 (0.1)	4 (0.2)	8 (0.4)	56 (2.8)	27 (1.4)	2 (0.1)	12 (0.6)	38 (1.9)	47 (2.4)	5 (0.3)	45 (2.3)	6 (0.3)	6 (0.3)	1 (0.1)	9 (0.5)	4 (0.2)	23 (1.2)
	*p*-value	**0.001**					**<0.001**				**0.021**							
Q3.	SA	10 (0.5)	28 (1.4)	40 (2.0)	160 (8.0)	36 (1.8)	4 (0.2)	30 (1.5)	162 (8.1)	78 (3.9)	14 (0.7)	125 (6.3)	12 (0.6)	15 (0.8)	4 (0.2)	20 (1.0)	9 (0.5)	75 (3.8)
	A	76 (3.8)	128 (6.4)	220 (11.0)	645 (32.3)	280 (14.0)	100 (5.0)	308 (15.4)	403 (20.2)	538 (26.9)	45 (2.3)	637 (31.9)	46 (2.3)	92 (4.6)	81 (4.1)	127 (6.4)	86 (4.3)	235 (11.8)
	U	2 (0.1)	4 (0.2)	4 (0.2)	24 (1.2)	14 (0.7)	0 (0.0)	10 (0.5)	14 (0.7)	22 (1.1)	2 (0.1)	21 (1.1)	2 (0.1)	3 (0.2)	4 (0.2)	1 (0.1)	2 (0.1)	11 (0.5)
	D	18 (0.9)	26 (1.3)	22 (1.1)	126 (6.3)	33 (1.7)	8 (0.4)	68 (3.4)	98 (4.9)	51 (2.6)	6 (0.3)	107 (5.4)	8 (0.4)	12 (0.6)	11 (0.5)	33 (1.7)	9 (0.5)	39 (2.0)
	SD	0 (0.0)	4 (0.2)	14 (0.7)	64 (3.2)	22 (1.1)	0 (0.0)	16 (0.8)	42 (2.1)	48 (2.4)	5 (0.3)	48 (2.4)	6 (0.3)	9 (0.5)	2 (0.1)	6 (0.3)	6 (0.3)	24 (1.2)
	*p*-value	**<0.001**					**<0.001**				**0.006**							
Q4.	SA	4 (0.2)	12 (0.6)	18 (0.9)	44 (2.2)	18 (0.9)	0 (0.0)	12 (0.6)	46 (2.3)	38 (1.9)	17 (0.9)	38 (1.9)	11 (0.5)	2 (0.1)	0 (0.0)	5 (0.3)	3 (0.2)	20 (1.0)
	A	28 (1.4)	20 (1.0)	28 (1.4)	132 (6.6)	38 (1.9)	18 (0.9)	104 (5.2)	64 (3.2)	60 (3.0)	19 (1.0)	104 (5.2)	22 (1.1)	10 (0.5)	18 (0.9)	27 (1.4)	11 (0.5)	35 (1.8)
	U	2 (0.1)	16 (0.8)	14 (0.7)	56 (2.8)	46 (2.3)	2 (0.1)	76 (3.8)	20 (1.0)	28 (1.4)	6 (0.3)	57 (2.9)	7 (0.4)	17 (0.9)	7 (0.4)	6 (0.3)	3 (0.2)	23 (01.2)
	D	72 (3.6)	132 (6.6)	238 (11.9)	749 (37.5)	273 (13.7)	92 (4.6)	228 (11.4)	533 (26.7)	611 (30.6)	29 (1.5)	706 (35.3)	33 (1.7)	101 (5.1)	77 (3.9)	144 (7.2)	86 (4.3)	288 (14.4)
	SD	0 (0.0)	10 (0.5)	2 (0.1)	38 (1.9)	10 (0.5)	0 (0.0)	12 (0.6)	56 (2.8)	0 (0.0)	1 (0.1)	33 (1.7)	1 (0.1)	1 (0.1)	0 (0.0)	5 (0.3)	9 (0.5)	18 (0.9)
	*p*-value	**<0.001**					**<0.001**				**<0.001**							
Q5.	SA	10 (0.5)	22 (1.1)	38 (1.9)	162 (8.1)	36 (1.8)	2 (0.1)	30 (1.5)	160 (8.0)	76 (3.8)	7 (0.4)	127 (6.4)	7 (0.4)	14 (0.7)	1 (0.1)	27 (1.4)	10 (0.5)	75 (3.8)
	A	78 (3.9)	134 (6.7)	232 (11.6)	665 (33.3)	285 (14.2)	98 (4.9)	322 (16.1)	437 (21.9)	537 (26.9)	43 (2.2)	664 (33.2)	42 (2.1)	99 (5.0)	82 (4.1)	125 (6.3)	91 (4.6)	248 (12.4)
	U	2 (0.1)	0 (0.0)	4 (0.2)	30 (1.5)	14 (0.7)	2 (0.1)	14 (0.7)	16 (0.8)	18 (0.9)	2 (0.1)	23 (1.2)	2 (0.1)	3 (0.2)	4 (0.2)	3 (0.2)	3 (0.2)	10 (0.5)
	D	14 (0.7)	24 (1.2)	16 (0.8)	118 (5.9)	30 (1.5)	2 (0.1)	62 (3.1)	66 (3.3)	72 (3.6)	16 (0.8)	85 (4.3)	18 (0.9)	8 (0.4)	12 (0.6)	28 (1.4)	2 (0.1)	33 (1.7)
	SD	2 (0.1)	10 (0.5)	10 (0.5)	44 (2.2)	20 (1.0)	8 (0.4)	4 (0.2)	16 (0.8)	34 (1.7)	4 (0.2)	39 (2.0)	5 (0.3)	7 (0.4)	3 (0.2)	4 (0.2)	6 (0.3)	18 (0.9)
	*p*-value	**<0.001**					**<0.001**				**<0.001**							
Q6.	SA	10 (0.5)	30 (1.5)	42 (2.1)	178 (8.9)	44 (2.2)	8 (0.4)	32 (1.6)	170 (8.5)	94 (4.7)	11 (0.5)	141 (7.0)	11 (0.5)	20 (1.0)	5 (0.3)	24 (1.2)	13 (0.7)	7 (4.0)
	A	76 (3.8)	122 (6.1)	220 (11.0)	625 (31.3)	274 (13.7)	96 (4.8)	320 (16.0)	399 (20.0)	502 (25.1)	39 (2.0)	629 (31.5)	37 (1.9)	90 (4.5)	83 (4.2)	127 (6.4)	83 (4.2)	229 (11.5)
	U	0 (0.0)	2 (0.1)	8 (0.4)	34 (1.7)	4 (0.2)	8 (0.4)	2 (0.1)	12 (0.6)	26 (1.3)	2 (0.1)	22 (1.1)	3 (0.2)	3 (0.2)	1 (0.1)	5 (0.3)	1 (0.1)	11 (0.5)
	D	18 (0.9)	24 (1.2)	18 (0.9)	122 (6.1)	47 (2.4)	0 (0.0)	62 (3.1)	96 (4.8)	71 (3.6)	16 (0.8)	99 (5.0)	18 (0.9)	12 (0.6)	10 (0.5)	25 (1.3)	8 (0.4)	41 (2.1)
	SD	2 (0.1)	12 (0.6)	12 (0.6)	60 (3.0)	16 (0.8)	0 (0.0)	16 (0.8)	42 (2.1)	44 (2.2)	4 (0.2)	47 (2.4)	5 (0.3)	6 (0.3)	3 (0.2)	6 (0.3)	7 (0.4)	24 (1.2)
	*p*-value	**<0.001**					**<0.001**				**0.001**							
Q7.	SA	2 (0.1)	8 (0.4)	10 (0.5)	28 (1.4)	16 (0.8)	4 (0.2)	4 (0.2)	30 (1.5)	26 (1.3)	4 (0.2)	28 (1.4)	5 (0.3)	5 (0.3)	2 (0.1)	2 (0.1)	4 (0.2)	14 (0.7)
	A	14 (0.7)	22 (1.1)	18 (0.9)	92 (4.6)	37 (1.9)	8 (0.4)	62 (3.1)	56 (2.8)	57 (2.9)	14 (0.7)	78 (3.9)	16 (0.8)	11 (0.5)	8 (0.4)	21 (1.1)	5 (0.3)	30 (1.5)
	U	2 (0.1)	0 (0.0)	12 (0.6)	48 (2.4)	22 (1.1)	2 (0.1)	40 (2.0)	18 (0.9)	24 (1.2)	3 (0.2)	39 (2.0)	4 (0.2)	5 (0.3)	10 (0.5)	6 (0.3)	4 (0.2)	13 (0.7)
	D	82 (4.1)	148 (7.4)	248 (12.4)	815 (40.8)	296 (14.8)	94 (4.7)	314 (15.7)	555 (27.8)	626 (31.3)	50 (2.5)	754 (37.7)	48 (2.4)	108 (5.4)	8 (0.4)	151 (7.6)	89 (4.9)	307 (15.4)
	SD	6 (0.3)	12 (0.6)	12 (0.6)	36 (1.8)	14 (0.7)	4 (0.2)	12 (0.6)	60 (3.0)	4 (0.2)	1 (0.1)	39 (2.0)	1 (0.1)	2 (0.1)	0 (0.0)	7 (0.4)	10 (0.5)	20 (1.0)
	*p*-value	**0.049**					**<0.001**				**<0.001**							
Q8.	SA	14 (0.7)	26 (1.3)	36 (1.8)	172 (8.6)	36 (1.8)	2 (0.1)	26 (1.3)	170 (8.5)	86 (4.3)	4 (0.2)	138 (6.9)	4 (0.2)	20 (1.0)	5 (0.3)	22 (1.1)	14 (0.7)	77 (3.9)
	A	66 (3.3)	116 (5.8)	230 (11.5)	633 (31.7)	288 (14.4)	92 (94.6)	330 (16.5)	393 (19.7)	518 (25.9)	47 (2.4)	629 (31.5)	46 (2.3)	91 (4.6)	78 (3.9)	122 (6.1)	85 (4.3)	235 (11.8)
	U	0 (0.0)	12 (0.6)	2 (0.1)	32 (1.6)	8 (0.4)	10 (0.5)	2 (0.1)	24 (1.2)	18 (0.9)	6 (0.3)	21 (1.1)	6 (0.3)	0 (0.0)	6 (0.3)	3 (0.2)	2 (0.1)	10 (0.5)
	D	24 (1.2)	28 (1.4)	22 (1.1)	126 (6.3)	33 (1.7)	8 (0.4)	66 (3.3)	82 (4.1)	77 (3.9)	11 (0.5)	106 (5.3)	13 (0.7)	13 (0.7)	11 (0.5)	33 (1.7)	6 (0.3)	40 (2.0)
	SD	2 (0.1)	8 (0.4)	10 (0.5)	56 (2.8)	20 (1.0)	0 (0.0)	8 (0.4)	50 (2.5)	38 (1.9)	4 (0.2)	44 (2.2)	5 (0.3)	7 (0.4)	2 (0.1)	7 (0.4)	5 (0.3)	22 (1.1)
	*p*-value	**<0.001**					**<0.001**				**<0.001**							
Q9.	SA	2 (0.1)	12 (0.6)	14 (0.7)	48 (2.4)	14 (0.7)	0 (0.0)	8 (0.4)	48 (2.4)	34 (1.7)	5 (0.3)	40 (2.0)	6 (0.3)	4 (0.2)	1 (0.1)	5 (0.3)	5 (0.3)	24 (1.2)
	A	16 (0.8)	34 (1.7)	16 (0.8)	166 (8.3)	47 (2.4)	10 (0.5)	88 (4.4)	82 (4.1)	99 (5.0)	25 (1.3)	122 (6.1)	20 (1.0)	16 (0.8)	14 (0.7)	35 (1.8)	10 (0.5)	37 (1.9)
	U	10 (0.5)	12 (0.6)	18 (0.9)	50 (2.5)	30 (1.5)	10 (0.5)	74 (3.7)	22 (1.1)	14 (0.7)	6 (0.3)	54 (2.7)	7 (0.4)	11 (0.5)	11 (0.5)	6 (0.3)	6 (0.3)	19 (1.0)
	D	76 (3.8)	120 (6.0)	248 (12.4)	717 (35.9)	284 (14.2)	92 (4.6)	254 (12.7)	517 (25.9)	582 (29.1)	36 (1.8)	689 (34.4)	41 (2.1)	99 (5.0)	76 (3.8)	133 (6.7)	83 (4.2)	288 (14.4)
	SD	2 (0.1)	12 (0.6)	4 (0.2)	38 (1.9)	10 (0.5)	0 (0.0)	8 (0.4)	50 (2.5)	8 (0.4)	0 (0.0)	33 (1.1)	0 (0.0)	1 (0.1)	0 (0.0)	8 (0.4)	8 (0.4)	16 (0.8)
	*p*-value	**<0.001**					**<0.001**				**<0.001**							
Q10.	SA	6 (0.3)	8 (0.45)	16 (0.8)	60 (3.0)	16 (0.8)	0 (0.0)	12 (0.6)	50 (2.5)	44 (2.2)	7 (0.4)	46 (2.3)	10 (0.5)	5 (0.3)	1 (0.1)	5 (0.3)	4 (0.2)	28 (1.4)
	A	14 (0.7)	18 (0.9)	18 (0.9)	100 (5.0)	32 (1.6)	6 (0.3)	68 (3.4)	58 (2.9)	50 (2.5)	11 (0.5)	80 (4.0)	12 (0.6)	10 (0.5)	11 (0.5)	27 (1.4)	4 (0.2)	27 (1.4)
	U	0 (0.0)	6 (0.3)	14 (0.7)	44 (2.2)	10 (0.5)	2 (0.1)	50 (2.5)	14 (0.7)	8 (0.4)	1 (0.1)	36 (1.8)	1 (0.1)	8 (0.4)	5 (0.3)	7 (0.4)	4 (0.2)	12 (0.6)
	D	78 (3.9)	142 (7.1)	246 (12.3)	771 (38.6)	307 (15.4)	102 (5.1)	292 (14.6)	531 (26.6)	619 (31.0)	52 (2.6)	730 (36.5)	50 (2.5)	104 (5.2)	84 (4.2)	140 (7.0)	88 (4.4)	296 (14.8)
	SD	8 (0.4)	16 (0.8)	6 (0.3)	44 (2.2)	20 (1.0)	2 (0.1)	10 (0.5)	66 (3.3)	16 (0.8)	1 (0.1)	46 (2.3)	1 (0.1)	4 (0.2)	1 (0.1)	8 (0.4)	12 (0.6)	21 (1.1)
	*p*-value	**0.020**					**<0.001**				**<0.001**							
Q11.	SA	8 (0.4)	18 (0.9)	32 (1.6)	144 (7.2)	24 (1.2)	2 (0.1)	26 (1.3)	146 (7.3)	52 (2.6)	1 (0.1)	112 (5.6)	1 (0.1)	9 (0.5)	3 (0.2)	22 (1.1)	10 (0.5)	68 (3.4)
	A	70 (3.5)	128 (6.4)	218 (10.9)	665 (33.3)	298 (14.9)	96 (4.8)	288 (14.4)	441 (22.1)	554 (27.7)	49 (2.5)	650 (32.55)	46 (2.3)	100 (5.0)	80 (4.0)	127 (6.4)	87 (4.4)	240 (12.0)
	U	4 (0.2)	12 (0.6)	16 (0.8)	48 (2.4)	10 (0.5)	8 (0.4)	32 (1.6)	28 (1.4)	22 (1.1)	4 (0.2)	41 (2.1)	6 (0.3)	4 (0.2)	7 (0.4)	7 (0.4)	4 (0.22)	17 (0.9)
	D	18 (0.9)	22 (1.1)	16 (0.8)	104 (5.2)	37 (1.9)	6 (0.3)	74 (3.7)	60 (3.0)	57 (2.9)	13 (0.7)	86 (4.3)	15 (0.8)	10 (0.5)	6 (0.3)	24 (1.2)	6 (0.3)	37 (1.9)
	SD	6 (0.3)	10 (0.5)	18 (0.9)	58 (2.9)	16 (0.8)	0 (0.0)	12 (0.6)	44 (2.2)	52 (2.6)	5 (0.3)	49 (2.5)	6 (0.3)	8 (0.4)	6 (0.3)	7 (0.4)	5 (0.3)	22 (1.1)
	*p*-value	**<0.001**					**<0.001**				**<0.001**							
Q12.	SA	6 (0.3)	8 (0.4)	12 (0.6)	60 (3.0)	18 (0.9)	0 (0.0)	8 (0.4)	46 (2.3)	50 (2.5)	5 (0.3)	47 (2.4)	8 (0.4)	5 (0.3)	3 (0.2)	6 (0.3)	4 (0.2)	26 (1.3)
	A	18 (0.9)	30 (1.5)	18 (0.9)	150 (7.5)	39 (2.0)	10 (0.5)	76 (3.8)	84 (4.2)	85 (4.3)	27 (1.4)	108 (5.4)	20 (1.0)	14 (0.7)	11 (0.5)	34 (1.7)	7 (0.4)	34 (1.4)
	U	4 (0.2)	8 (0.4)	10 (0.5)	34 (1.7)	18 (0.9)	6 (0.3)	40 (2.0)	16 (0.8)	12 (0.6)	2 (0.1)	35 (1.8)	3 (0.2)	7 (0.4)	5 (0.3)	1 (0.1)	5 (0.3)	16 (0.8)
	D	76 (3.8)	130 (6.5)	248 (12.4)	737 (36.9)	300 (15.0)	96 (4.8)	300 (15.0)	525 (26.3)	570 (28.5)	37 (1.9)	711 (35.6)	42 (2.1)	101 (5.1)	82 (4.1)	141 (7.0)	87 (4.4)	290 (14.5)
	SD	2 (0.1)	14 (0.7)	12 (0.6)	38 (1.9)	10 (0.5)	0 (0.0)	8 (0.4)	48 (2.4)	20 (1.0)	1 (0.1)	37 (1.9)	1 (0.1)	4 (0.2)	1 (0.1)	5 (0.3)	9 (0.5)	18 (0.9)
	*p*-value	**0.002**					**<0.001**				**<0.001**							
Q13.	SA	10 (0.5)	10 (0.5)	12 (0.6)	66 (3.3)	14 (0.7)	0 (0.0)	8 (0.4)	54 (2.7)	50 (2.5)	5 (0.3)	51 (2.6)	8 (0.4)	5 (0.3)	5 (0.3)	7 (0.4)	5 (0.3)	26 (1.3)
	A	12 (0.6)	22 (1.1)	28 (1.4)	124 (6.2)	29 (1.5)	10 (0.5)	72 (3.6)	68 (3.4)	65 (3.3)	13 (0.7)	95 (4.8)	13 (0.7)	13 (0.7)	10 (0.5)	30 (1.5)	7 (0.4)	34 (1.7)
	U	2 (0.1)	18 (0.9)	2 (0.1)	34 (1.7)	26 (1.3)	2 (0.1)	32 (1.6)	12 (0.6)	36 (1.8)	19 (1.7)	29 (1.9)	13 (0.7)	6 (0.3)	4 (0.2)	1 (0.1)	0 (0.0)	10 (0.5)
	D	80 (4.0)	128 (6.4)	246 (12.3)	743 (37.2)	302 (15.1)	100 (5.0)	312 (15.6)	529 (26.5)	558 (27.9)	34 (1.4)	718 (35.9)	39 (2.0)	103 (5.1)	82 (4.1)	142 (7.1)	90 (4.5)	291 (14.6)
	SD	2 (0.1)	12 (0.6)	12 (0.6)	52 (2.6)	14 (0.7)	0 (0.0)	8 (0.4)	56 (2.8)	28 (1.4)	1 (0.1)	45 (2.3)	1 (0.1)	4 (0.2)	1 (0.1)	7 (0.4)	10 (0.5)	23 (1.2)
	*p*-value	**<0.001**					**<0.001**				**<0.001**							
Q14.	SA	6 (0.3)	4 (0.2)	16 (0.8)	36 (1.8)	12 (0.6)	0 (0.0)	8 (0.4)	42 (2.1)	24 (1.2)	4 (0.2)	33 (1.7)	6 (0.3)	3 (0.2)	0 (0.0)	4 (0.2)	3 (0.2)	21 (1.1)
	A	10 (0.5)	20 (1.0)	12 (0.6)	100 (5.0)	16 (0.8)	4 (0.2)	64 (3.2)	46 (2.3)	44 (2.2)	11 (0.5)	68 (93.4)	12 (0.6)	6 (0.3)	9 (0.5)	25 (1.3)	3 (0.2)	24 (1.2)
	U	0 (0.0)	4 (0.2)	10 (0.5)	26 (1.3)	20 (1.0)	6 (0.3)	26 (1.3)	12 (0.6)	16 (0.8)	1 (0.1)	29 (1.5)	3 (0.2)	7 (0.4)	4 (0.2)	1 (0.1)	1 (0.1)	14 (0.7)
	D	86 (4.3)	144 (7.2)	250 (12.5)	807 (40.4)	313 (15.7)	94 (4.7)	326 (16.3)	551 (27.6)	629 (31.5)	55 (2.8)	755 (37.8)	52 (2.6)	109 (5.5)	88 (4.4)	147 (7.4)	93 (4.7)	301 (15.1)
	SD	4 (0.2)	18 (0.9)	12 (0.6)	50 (2.5)	24 (1.2)	8 (0.4)	8 (0.4)	68 (3.4)	24 (1.2)	1 (0.1)	53 (2.7)	1 (0.1)	6 (0.3)	1 (0.1)	10 (0.5)	12 (0.6)	24 (1.2)
	*p*-value	**<0.001**					**<0.001**				**<0.001**							
Q15.	SA	10 (0.5)	24 (1.2)	28 (1.4)	138 (6.9)	28 (1.4)	2 (0.1)	22 (1.1)	138 (6.9)	66 (3.3)	0 (0.0)	114 (5.7)	0 (0.0)	12 (0.6)	4 (0.2)	19 (1.0)	11 (0.5)	68 (3.4)
	A	64 (3.2)	110 (5.5)	204 (10.2)	503 (25.2)	252 (12.6)	74 (3.7)	166 (8.3)	409 (20.5)	484 (24.2)	40 (2.0)	536 (26.8)	35 (1.8)	74 (3.7)	60 (3.0)	113 (5.7)	76 (3.8)	199 (10.0)
	U	2 (0.1)	14 (0.7)	22 (1.1)	158 (7.9)	44 (2.2)	18 (0.9)	152 (7.6)	30 (1.5)	40 (2.0)	9 (0.5)	111 (5.6)	12 (0.6)	22 (1.1)	25 (1.3)	17 (0.9)	7 (0.4)	37 (1.9)
	D	20 (1.0)	34 (1.7)	30 (1.5)	146 (7.3)	40 (2.0)	14 (0.7)	76 (3.8)	94 (4.7)	86 (4.3)	18 (0.9)	117 (5.9)	21 (1.1)	14 (0.7)	9 (0.5)	30 (1.5)	10 (0.5)	51 (2.6)
	SD	10 (0.5)	8 (0.4)	16 (0.8)	74 (3.7)	21 (1.1)	4 (0.2)	16 (0.8)	48 (2.4)	61 (3.1)	5 (0.3)	60 (3.0)	6 (0.3)	9 (0.5)	4 (0.2)	8 (0.4)	8 (0.4)	29 (1.5)
	*p*-value	**<0.001**					**<0.001**				**<0.001**							
Q16.	SA	6 (0.3)	10 (0.5)	16 (0.8)	54 (2.7)	19 (1.0)	6 (0.3)	4 (0.2)	52 (2.6)	43 (2.2)	4 (0.2)	49 (2.5)	6 (0.3)	3 (0.2)	3 (0.2)	9 (0.5)	6 (0.3)	25 (1.3)
	A	14 (0.7)	32 (1.6)	12 (0.6)	126 (6.3)	50 (2.5)	4 (0.2)	80 (4.0)	76 (3.8)	74 (3.7)	14 (0.7)	103 (5.1)	15 (0.8)	17 (0.9)	14 (0.7)	27 (1.4)	7 (0.4)	37 (1.9)
	Un	6 (0.3)	0 (0.0)	4 (0.2)	20 (1.0)	0 (0.0)	6 (0.3)	6 (0.3)	14 (0.7)	4 (0.2)	2 (0.1)	13 (0.7)	3 (0.2)	0 (0.0)	2 (0.1)	2 (0.1)	1 (0.1)	7 (0.4)
	D	78 (3.9)	134 (6.7)	260 (13.0)	789 (39.5)	298 (14.9)	96 (4.8)	334 (16.7)	527 (26.4)	602 (30.1)	51 (2.6)	738 (36.9)	49 (2.5)	109 (5.5)	83 (4.2)	141 (7.0)	90 (4.5)	298 (14.9)
	SD	2 (0.1)	14 (0.7)	8 (0.4)	30 (1.5)	18 (0.9)	0 (0.0)	8 (0.4)	50 (2.5)	14 (0.7)	1 (0.1)	35 (1.8)	1 (0.1)	2 (0.1)	0 (0.0)	8 (0.4)	8 (0.4)	17 (0.9)
	*p*-value	**<0.001**					**<0.001**				**0.042**							
Q17.	SA	6 (0.3)	20 (1.0)	26 (1.3)	94 (4.7)	33 (1.7)	8 (0.4)	24 (1.2)	74 (3.7)	73 (3.7)	6 (0.3)	84 (4.2)	9 (0.5)	12 (0.6)	9 (0.5)	16 (0.8)	7 (0.4)	36 (1.8)
	A	16 (0.8)	18 (0.9)	8 (0.4)	114 (5.7)	26 (1.3)	2 (0.1)	72 (3.6)	68 (3.4)	40 (2.0)	10 (0.5)	81 (4.1)	10 (0.5)	10 (0.5)	6 (0.3)	27 (1.4)	8 (0.4)	30 (1.5)
	U	0 (0.0)	0 (0.0)	0 (0.0)	10 (0.5)	0 (0.0)	0 (0.0)	4 (0.2)	6 (0.3)	8 (0.4)	0 (0.0)	9 (0.5)	0 (0.0)	2 (0.1)	1 (0.1)	0 (0.0)	0 (0.0)	6 (0.3)
	D	76 (3.8)	122 (6.1)	224 (11.2)	114 (5.7)	269 (13.5)	88 (4.4)	320 (16.0)	483 (24.2)	489 (24.5)	56 (2.8)	643 (32.2)	55 (2.8)	95 (4.8)	69 (3.5)	120 (6.0)	72 (3.6)	270 (13.5)
	SD	8 (0.4)	30 (1.5)	42 (2.1)	112 (5.6)	49 (2.5)	14 (0.7)	12 (0.6)	88 (4.4)	127 (6.4)	0 (0.0)	121 (6.1)	0 (0.0)	12 (0.6)	17 (0.9)	24 (1.2)	25 (1.3)	42 (2.1)
	*p*-value	**<0.001**					**<0.001**				**0.002**							
Q18.	SA	12 (0.6)	26 (1.3)	42 (2.1)	150 (7.5)	38 (1.9)	6 (0.3)	30 (1.5)	160 (8.0)	72 (3.6)	2 (0.1)	132 (6.6)	2 (0.1)	19 (1.0)	2 (0.1)	25 (1.3)	12 (0.6)	74 (3.7)
	A	72 (3.6)	122 (6.1)	226 (11.3)	663 (33.2)	280 (14.0)	96 (4.8)	322 (16.1)	425 (21.3)	520 (26.0)	49 (2.5)	642 (32.1)	48 (2.4)	89 (4.5)	87 (4.4)	121 (6.1)	89 (4.5)	238 (11.9)
	U	0 (0.0)	4 (0.2)	2 (0.1)	16 (0.8)	16 (0.8)	0 (0.0)	12 (0.6)	8 (0.4)	18 (0.9)	2 (0.1)	17 (0.9)	2 (0.1)	4 (0.2)	1 (0.1)	3 (0.2)	0 (0.0)	9 (0.5)
	D	20 (1.0)	30 (1.5)	20 (1.0)	138 (6.9)	37 (1.9)	10 (0.5)	64 (3.2)	78 (3.9)	93 (4.7)	15 (0.8)	108 (5.4)	17 (0.9)	15 (0.8)	10 (0.5)	29 (1.5)	7 (0.4)	44 (2.2)
	SD	2 (0.1)	8 (0.4)	10 (0.5)	52 (2.6)	14 (0.7)	0 (0.0)	4 (0.2)	48 (2.4)	34 (1.7)	4 (0.2)	39 (2.0)	5 (0.3)	4 (0.2)	2 (0.1)	9 (0.5)	4 (0.2)	19 (1.0)
	*p*-value	**<0.001**					**<0.001**				**<0.001**							

* SA, Strongly Agree; A, Agree; U, Uncertain; D, Disagree; SD, Strongly Disagree; AP, Arunachal Pradesh; As, Assam; Mn, Manipur; Mg, Meghalaya; Mz, Mizoram; Ng, Nagaland; Sk, Sikkim; Tr, Tripura. Bold values are indicates all the statistically significant *p* values.

**Table 7:** Bivariate analysis: examining the impact of hospital type and stay duration on patient satisfaction.*

Questions (Item numbers) (see Appendix)		Type of hospital			Duration of stay in the hospital				
		Government	Private	Semi-government	<1 week	1 week	2 weeks	3 weeks	>3 weeks
Q1.	SA	186 (9.3)	65 (3.3)	10 (0.5)	54 (2.7)	75 (3.8)	84 (4.2)	44 (2.2)	4 (0.2)
	A	863 (43.2)	485 (24.3)	34 (1.7)	536 (26.8)	359 (18.0)	248 (12.4)	185 (9.3)	54 (2.7)
	U	26 (1.3)	14 (0.7)	0 (0.0)	12 (0.6)	8 (0.4)	14 (0.7)	4 (0.2)	2 (0.1)
	D	128 (6.4)	64 (3.2)	10 (0.5)	74 (3.7)	50 (2.5)	22 (1.1)	46 (2.35)	10 (0.5)
	SD	101 (5.1)	12 (0.6)	2 (0.1)	39 (2.0)	30 (1.5)	32 (1.6)	14 (0.7)	0 (0.0)
	*p*-value	**<0.001**			**<0.001**				
Q2.	SA	200 (10.0)	62 (3.1)	10 (0.5)	72 (3.6)	72 (3.6)	82 (4.1)	42 (2.1)	4 (0.2)
	A	837 (41.9)	512 (25.6)	36 (1.8)	522 (26.1)	368 (18.4)	252 (12.6)	189 (9.5)	54 (2.7)
	U	40 (2.0)	4 (0.2)	0 (0.0)	14 (0.7)	10 (0.5)	14 (0.7)	2 (0.1)	4 (0.2)
	D	136 (6.8)	56 (2.8)	8 (0.4)	68 (3.4)	50 (2.5)	30 (1.5)	44 (2.2)	8 (0.4)
	SD	91 (4.6)	6 (0.3)	2 (0.1)	39 (2.0)	22 (1.1)	22 (1.1)	16 (0.8)	0 (0.0)
	*p*-value	**<0.001**			**<0.001**				
Q3.	SA	204 (10.2)	54 (2.7)	16 (0.8)	78 (3.9)	70 (3.5)	84 (4.2)	38 (1.9)	4 (0.2)
	A	811 (40.6)	508 (25.4)	30 (1.5)	504 (25.2)	368 (18.4)	236 (11.8)	185 (9.3)	56 (2.8)
	U	38 (1.9)	8 (0.4)	0 (0.0)	16 (0.8)	10 (0.5)	12 (0.6)	4 (0.2)	4 (0.2)
	D	157 (7.9)	60 (3.0)	8 (0.4)	81 (4.1)	50 (2.5)	36 (1.8)	52 (2.6)	6 (0.3)
	SD	94 (4.7)	10 (0.5)	2 (0.1)	36 (1.8)	24 (1.2)	32 (1.6)	14 (0.7)	0 (0.0)
	*p*-value	**<0.001**			**<0.001**				
Q4.	SA	82 (4.1)	12 (0.6)	2 (0.1)	26 (1.3)	30 (1.5)	20 (1.0)	20 (1.0)	0 (0.0)
	A	158 (7.9)	76 (3.8)	12 (0.6)	62 (3.1)	86 (4.3)	44 (2.2)	50 (2.5)	4 (0.2)
	U	90 (4.5)	32 (1.6)	4 (0.2)	40 (2.0)	42 (2.1)	22 (1.1)	20 (1.0)	2 (0.1)
	D	936 (46.8)	496 (24.8)	32 (1.6)	581 (29.1)	350 (17.5)	280 (14.0)	191 (9.6)	62 (3.1)
	SD	38 (1.9)	24 (1.2)	6 (0.3)	6 (0.3)	14 (0.7)	34 (1.7)	12 (0.6)	2 (0.1)
	*p*-value	**<0.001**			**<0.001**				
Q5.	SA	198 (9.9)	52 (2.6)	18 (0.9)	60 (3.0)	72 (3.6)	86 (4.3)	46 (2.3)	4 (0.2)
	A	866 (43.3)	502 (35.0)	28 (1.4)	525 (26.3)	366 (18.3)	250 (12.5)	199 (10.0)	54 (2.7)
	U	38 (1.9)	10 (0.5)	2 (0.1)	18 (0.9)	12 (0.6)	14 (0.7)	2 (0.1)	4 (0.2)
	D	128 (6.4)	68 (3.4)	6 (0.3)	70 (3.5)	56 (2.8)	30 (1.5)	38 (1.9)	8 (0.4)
	SD	74 (3.7)	10 (0.5)	2 (0.1)	42 (2.1)	16 (0.8)	20 (1.0)	8 (0.4)	0 (0.0)
	*p*-value	**<0.001**			**<0.001**				
Q6.	SA	210 (10.5)	78 (3.9)	16 (0.8)	86 (4.3)	76 (3.8)	90 (4.5)	44 (2.2)	8 (0.4)
	A	821 (41.1)	470 (23.5)	26 (1.3)	492 (24.6)	360 (18.0)	236 (11.8)	177 (8.9)	52 (2.6)
	U	28 (1.4)	14 (0.7)	6 (0.3)	20 (1.0)	8 (0.4)	12 (0.6)	4 (0.2)	4 (0.2)
	D	157 (7.9)	64 (3.2)	8 (0.4)	73 (3.7)	66 (3.3)	34 (1.7)	50 (2.5)	6 (0.3)
	SD	88 (4.4)	14 (0.7)	16 (0.8)	44 (2.8)	12 (0.6)	28 (1.4)	18 (0.9)	0 (0.0)
	*p*-value	**<0.001**			**<0.001**				
Q7.	SA	52 (2.6)	10 (0.5)	2 (0.1)	28 (1.4)	16 (0.8)	14 (0.7)	6 (0.3)	0 (0.0)
	A	99 (5.0)	74 (3.7)	10 (0.5)	65 (3.3)	54 (2.7)	30 (91.5)	30 (1.5)	4 (0.2)
	U	62 (3.1)	20 (1.0)	2 (0.1)	36 (1.8)	20 (1.0)	22 (1.1)	0 (0.0)	2 (0.1)
	D	1,045 (52.3)	508 (25.4)	36 (1.8)	578 (28.9)	408 (20.4)	308 (15.4)	2,379 (11.9)	58 (2.9)
	SD	46 (2.3)	28 (1.4)	6 (0.3)	8 (0.4)	24 (1.2)	26 (1.3)	20 (1.0)	6 (0.3)
	*p*-value	**<0.001**			**<0.001**				
Q8.	SA	188 (9.4)	78 (3.9)	18 (0.9)	80 (4.0)	70 (3.5)	82 (4.1)	44 (2.2)	8 (0.4)
	A	835 (41.8)	472 (23.6)	26 (1.3)	478 (23.9)	364 (18.2)	256 (12.8)	185 (9.3)	50 (2.5)
	U	36 (1.8)	18 (0.9)	0 (0.0)	26 (1.3)	14 (0.7)	8 (0.4)	0 (0.0)	6 (0.3)
	D	159 (8.0)	64 (3.2)	10 (0.5)	89 (4.5)	54 (2.7)	32 (1.6)	52 (2.6)	6 (0.3)
	SD	86 (4.3)	8 (0.4)	2 (0.1)	42 (2.1)	20 (1.0)	22 (1.1)	12 (0.6)	0 (0.0)
	*p*-value	**<0.001**			**<0.001**				
Q9.	SA	78 (3.9)	10 (0.5)	2 (0.1)	36 (1.8)	22 (1.1)	24 (1.2)	8 (0.4)	0 (0.0)
	A	185 (9.3)	86 (4.3)	8 (0.4)	99 (5.0)	76 (3.8)	36 (1.8)	62 (3.1)	6 (0.3)
	U	108 (5.4)	12 (0.6)	0 (0.0)	36 (1.8)	44 (2.2)	28 (1.4)	6 (0.3)	6 (0.3)
	D	893 (44.7)	512 (25.6)	40 (2.0)	540 (27.0)	362 (18.1)	288 (14.4)	199 (10.0)	56 (2.8)
	SD	40 (2.0)	20 (1.0)	6 (0.3)	4 (0.2)	18 (0.9)	24 (1.2)	18 (0.9)	2 (0.1)
	*p*-value	**<0.001**			**<0.001**				
Q10.	SA	88 (4.4)	14 (0.7)	4 (0.2)	40 (2.0)	28 (1.4)	22 (1.1)	14 (0.7)	2 (0.1)
	A	114 (5.7)	62 (3.1)	6 (0.3)	62 (3.1)	52 (2.6)	30 (1.5)	36 (1.8)	2 (0.1)
	U	60 (3.0)	14 (0.7)	0 (0.0)	28 (1.4)	22 (1.1)	14 (0.7)	8 (0.4)	2 (0.1)
	D	1,000 (50.0)	504 (25.2)	40 (2.0)	557 (27.9)	402 (20.1)	306 (15.3)	221 (11.1)	58 (2.9)
	SD	42 (2.1)	46 (2.3)	6 (0.3)	28 (1.4)	18 (0.9)	28 (1.4)	14 (0.7)	6 (0.3)
	*p*-value	**<0.001**			0.206				
Q11.	SA	160 (8.0)	52 (2.6)	14 (0.7)	54 (2.7)	54 (2.7)	78 (3.9)	34 (1.7)	6 (0.3)
	A	877 (43.9)	476 (23.8)	26 (1.3)	512 (25.6)	370 (18.5)	244 (12.2)	197 (9.9)	56 (2.8)
	U	58 (2.9)	24 (1.2)	8 (0.4)	30 (1.5)	30 (1.5)	18 (0.9)	12 (0.6)	0 (0.0)
	D	121 (6.1)	68 (3.4)	8 (0.4)	63 (3.2)	52 (2.6)	34 (1.7)	40 (2.0)	8 (0.4)
	SD	88 (4.4)	20 (1.0)	0 (0.0)	56 (2.8)	16 (0.8)	26 (1.3)	10 (0.5)	0 (0.0)
	*p*-value	**<0.001**			**<0.001**				
Q12.	SA	9 (4.5)	8 (0.4)	6 (0.3)	34 (1.7)	34 (1.7)	22 (1.1)	12 (0.6)	2 (0.1)
	A	173 (8.6)	78 (3.9)	4 (0.2)	99 (5.0)	64 (3.2)	34 (1.7)	54 (2.7)	4 (0.2)
	U	58 (2.9)	16 (0.8)	0 (0.0)	18 (0.9)	28 (1.4)	22 (1.1)	4 (0.2)	2 (0.1)
	D	937 (46.9)	514 (25.7)	40 (2.0)	550 (27.5)	378 (18.9)	294 (14.7)	209 (10.5)	60 (3.0)
	SD	46 (2.3)	24 (1.2)	6 (0.3)	14 (0.7)	18 (0.9)	28 (1.4)	14 (0.7)	2 (0.1)
	*p*-value	**<0.001**			**<0.001**				
Q13.	SA	96 (4.8)	12 (0.6)	4 (0.2)	46 (2.3)	26 (1.3)	24 (1.2)	14 (0.7)	2 (0.1)
	A	143 (7.2)	62 (3.1)	10 (0.5)	77 (3.9)	50 (2.5)	34 (1.7)	50 (2.5)	4 (0.2)
	U	62 (3.1)	20 (1.0)	0 (0.0)	26 (1.3)	42 (2.1)	6 (0.3)	8 (0.4)	0 (0.0)
	D	945 (47.3)	518 (25.9)	36 (1.8)	538 (26.9)	382 (19.1)	310 (15.5)	207 (10.4)	62 (3.1)
	SD	58 (2.9)	28 (1.4)	6 (0.3)	28 (1.4)	22 (1.1)	26 (1.3)	14 (0.7)	2 (0.1)
	*p*-value	**<0.001**			**<0.001**				
Q14.	SA	66 (3.3)	4 (0.2)	4 (0.2)	18 (0.9)	16 (0.8)	26 (1.3)	12 (0.6)	2 (0.1)
	A	102 (5.1)	52 (2.6)	4 (0.2)	56 (2.8)	34 (1.7)	22 (1.1)	42 (2.1)	4 (0.2)
	U	34 (1.7)	20 (1.0)	6 (0.3)	22 (1.1)	30 (1.5)	4 (0.2)	2 (0.1)	2 (0.1)
	D	1,042 (52.1)	522 (26.1)	36 (1.8)	579 (29.0)	422 (21.1)	316 (15.8)	223 (11.2)	60 (3.0)
	SD	60 (3.0)	42 (2.1)	6 (0.3)	40 (2.0)	20 (1.0)	32 (1.6)	14 (0.7)	2 (0.1)
	*p*-value	**<0.001**			**<0.001**				
Q15.	SA	166 (8.3)	50 (2.5)	12 (0.6)	56 (2.8)	60 (3.0)	76 (3.8)	34 (1.7)	2 (0.1)
	A	675 (33.8)	430 (21.5)	28 (1.4)	456 (22.8)	254 (12.7)	204 (10.2)	169 (8.5)	50 (2.5)
	U	190 (9.5)	48 (2.4)	2 (0.1)	58 (2.9)	108 (5.4)	44 (2.2)	20 (1.0)	10 (0.5)
	D	164 (8.2)	92 (4.6)	14 (0.7)	88 (4.4)	76 (3.8)	48 (2.4)	50 (2.5)	8 (0.4)
	SD	109 (5.5)	20 (1.0)	0 (0.0)	57 (2.9)	24 (1.2)	28 (1.4)	20 (1.0)	0 (0.0)
	*p*-value	**<0.001**			**<0.001**				
Q16.	SA	87 (4.4)	14 (0.7)	4 (0.2)	43 (2.2)	22 (1.1)	26 (1.3)	12 (0.6)	2 (0.1)
	A	154 (7.7)	74 (3.7)	6 (0.3)	88 (4.4)	60 (3.0)	32 (1.6)	48 (2.4)	6 (0.3)
	U	26 (1.3)	4 (0.2)	0 (0.0)	14 (7.0)	12 (0.6)	4 (0.2)	0 (0.0)	0 (0.0)
	D	999 (50.0)	520 (26.0)	40 (2.0)	552 (27.6)	414 (20.7)	314 (15.7)	219 (11.0)	60 (3.0)
	SD	38 (1.9)	28 (1.4)	6 (0.3)	18 (0.9)	14 (0.7)	24 (1.2)	14 (0.7)	2 (0.1)
	*p*-value	**<0.001**			**0.002**				
Q17.	SA	141 (7.0)	32 (1.6)	6 (0.3)	81 (4.1)	40 (2.0)	40 (2.0)	14 (0.7)	4 (0.2)
	A	118 (5.9)	60 (3.0)	4 (0.2)	42 (2.1)	48 (2.4)	32 (1.6)	58 (2.9)	2 (0.1)
	U	12 (0.6)	6 (0.3)	0 (0.0)	6 (0.3)	6 (0.3)	2 (0.1)	0 (0.0)	4 (0.2)
	D	892 (44.6)	45 (22.5)	38 (1.9)	484 (24.2)	372 (18.6)	282 (14.1)	190 (9.5)	52 (2.6)
	SD	141 (7.0)	92 (4.6)	8 (0.4)	102 (5.1)	56 (2.8)	44 (2.2)	31 (1.6)	8 (0.4)
	*p*-value	**0.005**			**<0.001**				
Q18	SA	182 (9.1)	72 (3.6)	14 (0.7)	78 (3.9)	62 (3.1)	86 (4.3)	36 (1.8)	6 (0.3)
	A	851 (42.6)	480 (24.0)	32 (1.6)	500 (25.0)	364 (18.2)	248 (12.4)	195 (9.8)	56 (2.8)
	U	32 (1.6)	6 (0.3)	0 (0.0)	16 (0.8)	6 (0.3)	10 (0.5)	4 (0.2)	2 (0.1)
	D	159 (8.0)	76 (3.8)	10 (0.5)	81 (4.1)	78 (3.9)	34 (1.7)	46 (2.3)	6 (0.3)
	SD	80 (4.0)	6 (0.3)	0 (0.0)	40 (2.0)	12 (0.6)	22 (1.1)	12 (0.6)	0 (0.0)
	*p*-value	**<0.001**			**<0.001**				

* SA, Strongly Agree; A, Agree; U, Uncertain; D, Disagree; SD, Strongly Disagree. Bold values are indicates all the statistically significant *p* values.

## 4 Discussion

The results of this study provide valuable insights into the demographics, health-related variables, COVID-19 vaccination status, healthcare settings, post-COVID-19 complications, and patient satisfaction among participants in Northeast India. In the forthcoming discussion, the implications and significance of these findings are discussed.

The study’s diverse sample population, comprising a large portion of the participants in the 26–33 age bracket, with fairly balanced gender distribution, revealed nuanced insights into patient satisfaction. A significant proportion of married participants had distinct healthcare expectations compared to single individuals or divorced/widowed participants. A predominant number of the study participants identified as Hindus, followed by Muslims, which illustrated the cultural diversity that might have influenced healthcare practices and preferences (8). Education levels varied, with graduates forming the largest group and a smaller but significant portion being illiterate or uneducated, potentially impacting health literacy and healthcare decision-making (9). The occupation mix, including healthcare workers and others, suggested differences in healthcare perspectives, while the predominance of participants from the private sector highlighted potential variations in healthcare access and coverage compared to government and self-employed individuals (10). The household income distribution, with a substantial portion earning 2.6 lac and above, indicated potential disparities in affordability and access to healthcare services (11). These socio-demographic variables collectively contribute to a comprehensive understanding of how patient satisfaction may vary across distinct demographic groups within Northeast India’s population.

The study’s findings indicated a noteworthy and positive trend in COVID-19 vaccination among participants in Northeast India, with a substantial majority having received the vaccine, both the first and the second dose. This high vaccination rate of the study participants underscored the success of widespread vaccination efforts in the region, contributing to community-level immunity against the virus (12). However, it is equally vital to examine the reasons behind the comparatively lesser number of those participants who had not received their first dose and the second dose. The data revealed that reasons for non-vaccination included concerns about perceived need, fear of side effects, vaccine unavailability, medical conditions, and a lack of awareness about the vaccine. Addressing these concerns and barriers is imperative to further enhance vaccination rates and ensure that a larger proportion of the population is protected against COVID-19. By understanding these reasons, public health strategies can be refined to provide targeted information, resources, and support to address hesitancy and improve vaccine accessibility, ultimately contributing to the region’s overall health and safety.

The study’s findings on post-COVID-19 complications underscored the diverse and multi-faceted health impacts experienced by the COVID-19 survivors in Northeast India. Notably, participants reported a range of complications, with a significant number of those reporting no post-COVID-19 complications, indicating that a substantial portion of survivors did not experience additional health issues after recovering from the virus. However, the data also revealed specific complications affecting some participants, including acute cardiac injury, acute respiratory failure, anxiety and depression, asthma, breathlessness, and chest pain, chronic fatigue, excessive weakness, weight loss, GI problems, pneumonia, and loss of smell. These findings highlighted the need for healthcare providers to be prepared to address a wide range of post-COVID-19 complications, providing tailored care and support to those who require it (13). Additionally, ongoing research and monitoring of these complications can contribute to a better understanding of the long-term health effects of COVID-19 and inform strategies for comprehensive post-recovery care in the region.

The distribution of participants across various healthcare settings provided valuable insights into the accessibility and utilization of healthcare services in Northeast India. Notably, a vast number of the participants received treatment in government hospitals, which suggested a significant reliance on government healthcare facilities, possibly influenced by factors like affordability and perceived quality of care (14). Additionally, the study found a notable variation in the duration of hospital stays, with majority staying for less than 1 week, and only a small percentage of the participants stayed for more than 3 weeks. These varying hospitals stay durations can be attributed to the severity of COVID-19 cases, the healthcare infrastructure’s capacity, and individual patient needs. Understanding the factors driving these choices and durations is vital for healthcare planning and resource allocation, ensuring that healthcare services can effectively cater to the diverse needs of the population while maintaining high-quality care and accessibility.

The analysis of patient satisfaction using the PSQ-18 questionnaire offered a comprehensive understanding of the healthcare experiences of participants in Northeast India with a large number of the participants expressing overall high satisfaction, with positive perceptions in areas such as doctor-patient communication and medical care quality where most of the participants agreed or strongly agreed with the provided statements. This finding reflected the commendable efforts of healthcare providers in delivering effective and informative care. However, areas of concern emerged, including financial aspects and long wait times for emergency treatment, where most of the participants disagreed or strongly disagreed. These findings highlighted the need for strategies to address financial barriers and reduce wait times to enhance overall satisfaction. Additionally, some participants reported feeling that doctors were impersonal or ignored their concerns, indicating room for improvement in doctor-patient interactions. Strengthening these interactions can contribute to a more holistic and patient-centered healthcare experience, ultimately fostering higher satisfaction levels among COVID-19 survivors in the region (15).

The study’s analysis of factors influencing patient satisfaction, including education status, household income, and states of residence, offered valuable insights into the determinants of healthcare experiences among COVID-19 survivors in Northeast India. On examining the data, it became evident that these demographic and regional factors significantly correlated with satisfaction levels. For instance, participants with higher education levels reported higher satisfaction scores, with those in the “graduate” category notably comprising the majority. Similarly, household income played a role, with a substantial portion reporting incomes of 2.6 lac and above expressing higher satisfaction levels. Additionally, variations across states of residence were observed, suggesting that regional differences may influence patient satisfaction. These results reflect the necessity of customizing healthcare policies and services to accommodate the distinct requirements and preferences of various demographic groups and regions, with the ultimate goal of enhancing healthcare experiences in an equitable manner for all.

The consistently high levels of patient satisfaction observed across various hospital types and lengths of hospital stays in Northeast India was a reassuring finding. It suggested that patients tend to receive satisfactory care irrespective of whether they seek treatment in government hospitals, private hospitals, or semi-government hospitals. Furthermore, the diverse duration of hospital stays demonstrated that patients experience high satisfaction levels regardless of how long they are hospitalized. These results indicate that healthcare quality is maintained consistently across different healthcare settings and durations of care, emphasizing the region’s commitment to providing satisfactory healthcare services to its population, regardless of where and for how long treatment is sought.

Based on these findings, several implications for healthcare policy and practices can be drawn:

**Improving Doctor-Patient Interactions**: Efforts should be made to enhance doctor-patient communication and address patient concerns to improve overall satisfaction. Training programs for healthcare providers in interpersonal skills and empathy may be beneficial.

**Addressing Financial Concerns**: Strategies to make healthcare more affordable and reduce financial burdens on patients, such as insurance schemes or subsidies, could be explored.

**Vaccination Awareness and Accessibility**: To further increase vaccination rates, awareness campaigns should address concerns about vaccine safety and availability.

**Tailoring Services**: Healthcare services can be tailored to meet the specific needs of different demographic groups and regions, considering education, income, and state of residence.

**Continued Monitoring**: Regular monitoring of patient satisfaction and healthcare quality is essential to identify areas for improvement and track the impact of healthcare interventions.

## 5 Limitations

Despite its valuable insights, the study acknowledges limitations that warrant consideration. Recall bias could have influenced participants’ recollection of specific details, potentially impacting the accuracy of self-reported information. The sampling strategy, concentrated in specific regions, may have introduced sampling bias, limiting generalizability to the entire Northeast Indian population. Additionally, the demographic homogeneity might not sufficiently represent the region’s diverse demographics. Finally, relying solely on self-reported data introduces the possibility of inaccurate responses due to social desirability bias or incomplete understanding of medical terminology. Addressing these limitations in future research is crucial for refining future understanding of healthcare experiences in Northeast India.

## 6 Conclusion

The study emphasized the need for tailored healthcare policies and services in Northeast India, given the diverse demographic factors that influence patient satisfaction. The high vaccination rate signifies the success of public health efforts, yet understanding vaccine hesitancy remains crucial. Similarly, while many reported overall satisfaction, concerns regarding affordability, wait times, and doctor-patient communication demand attention. Financial hardships and impersonal interactions can significantly impact patient experiences. The study also highlights the importance of tailoring healthcare services to diverse demographic groups. Education, income, and regional variations influence satisfaction, necessitating targeted interventions. Addressing the needs of the less educated, lower-income populations, and residents of specific states becomes pivotal. The diverse range of post-COVID complications underscores the need for comprehensive care beyond recovery. Healthcare providers must be prepared to manage long-term effects, while ongoing research offers valuable insights into these complexities. These findings call for a collaborative approach involving policymakers, healthcare providers, and communities. Training programs to enhance doctor-patient interactions, financial assistance schemes, targeted awareness campaigns, and tailored healthcare services are necessary steps toward a more equitable and patient-centered healthcare system. Regular monitoring of patient satisfaction and healthcare quality is vital to ensure sustained progress. By acting on these insights, stakeholders can empower individuals in Northeast India to navigate the healthcare landscape with confidence and experience the full potential of quality care.

## Data availability statement

The raw data supporting the conclusions of this article will be made available by the authors, without undue reservation.

## Ethics statement

The studies involving humans were approved by University of Amity Institutional Review Board (IRB No. AUUP/IEC/MAY/2023/4). The studies were conducted in accordance with the local legislation and institutional requirements. The participants provided their written informed consent to participate in this study.

## Author contributions

SS: Conceptualization, Writing – original draft. FN: Methodology, Writing – original draft. KK: Formal analysis, Writing – original draft. SR: Writing – review & editing. MS: Writing – review & editing. HS: Supervision, Writing – review & editing.

## Appendix 1

**Question (Item numbers)**.

Q1. Doctors are good about explaining the reason for medical tests.
Q2. I think my doctor’s office has everything needed to provide complete medical care.
Q3. The medical care I have been receiving is just about perfect.
Q4. Sometimes doctors make me wonder if their diagnosis is correct.
Q5. I feel confident that I can get the medical care I need without being set back financially.
Q6. When I go for medical care, they are careful to check everything when treating and examining me.
Q7. I have to pay for more of my medical care than I can afford.
Q8. I have easy access to the medical specialists I need.
Q9. Where I get medical care, people have to wait too long for emergency treatment.
Q10. Where I get medical care, people have to wait too long for emergency treatment.
Q11. My doctors treat me in a very friendly and courteous manner.
Q12. Those who provide my medical care sometimes hurry too much when they treat me.
Q13. Doctors sometimes ignore what I tell them.
Q14. I have some doubts about the ability of the doctors who treat me.
Q15. Doctors usually spend plenty of time with me.
Q16. I find it hard to get an appointment for medical care right away.
Q17. I am dissatisfied with some things about the medical care I receive.
Q18. I am able to get medical care whenever I need it.
